# Cardiac-derived CTRP9 protects against myocardial ischemia/reperfusion injury via calreticulin-dependent inhibition of apoptosis

**DOI:** 10.1038/s41419-018-0726-3

**Published:** 2018-06-20

**Authors:** Dajun Zhao, Pan Feng, Yang Sun, Zhigang Qin, Zhengbin Zhang, Yanzhen Tan, Erhe Gao, Wayne Bond Lau, Xinliang Ma, Jian Yang, Shiqiang Yu, Xuezeng Xu, Dinghua Yi, Wei Yi

**Affiliations:** 1 Department of Cardiovascular Surgery, Xijing Hospital, Fourth Military Medical University, 710032 Xi’an, China; 2 Department of Geriatric, Xijing Hospital, Fourth Military Medical University, 710032 Xi’an, China; 3 Center for Translational Medicine, Temple University, Philadelphia, PA 19140 USA; 4 Department of Emergency Medicine, Thomas Jefferson University, Philadelphia, PA 19107 USA

## Abstract

Cardiokines play an essential role in maintaining normal cardiac functions and responding to acute myocardial injury. Studies have demonstrated the heart itself is a significant source of C1q/TNF-related protein 9 (CTRP9). However, the biological role of cardiac-derived CTRP9 remains unclear. We hypothesize cardiac-derived CTRP9 responds to acute myocardial ischemia/reperfusion (MI/R) injury as a cardiokine. We explored the role of cardiac-derived CTRP9 in MI/R injury via genetic manipulation and a CTRP9-knockout (CTRP9-KO) animal model. Inhibition of cardiac CTRP9 exacerbated, whereas its overexpression ameliorated, left ventricular dysfunction and myocardial apoptosis. Endothelial CTRP9 expression was unchanged while cardiomyocyte CTRP9 levels decreased after simulated ischemia/`reperfusion (SI/R) in vitro. Cardiomyocyte CTRP9 overexpression inhibited SI/R-induced apoptosis, an effect abrogated by CTRP9 antibody. Mechanistically, cardiac-derived CTRP9 activated anti-apoptotic signaling pathways and inhibited endoplasmic reticulum (ER) stress-related apoptosis in MI/R injury. Notably, CTRP9 interacted with the ER molecular chaperone calreticulin (CRT) located on the cell surface and in the cytoplasm of cardiomyocytes. The CTRP9–CRT interaction activated the protein kinase A-cAMP response element binding protein (PKA-CREB) signaling pathway, blocked by functional neutralization of the autocrine CTRP9. Inhibition of either CRT or PKA blunted cardiac-derived CTRP9’s anti-apoptotic actions against MI/R injury. We further confirmed these findings in CTRP9-KO rats. Together, these results demonstrate that autocrine CTRP9 of cardiomyocyte origin protects against MI/R injury via CRT association, activation of the PKA-CREB pathway, ultimately inhibiting cardiomyocyte apoptosis.

## Introduction

Ischemic heart disease (IHD) is the leading cause of death and disability worldwide^1,2^. Cardiokines are a group of proteins present in secretomes produced by the heart^3,4^. They maintain cardiac homeostasis and modulate pathological remodeling in response to stress via autocrine/paracrine pathways^5^. Some cardiokines are secreted during ischemic stress, and function to salvage viable myocardial structure and function via anti-apoptotic and anti-inflammatory properties^6–9^. These endogenous cardiokines may serve as novel therapeutic targets against IHD, given their immediate response to acute myocardial injury^3^.

C1q/TNF-related protein 9 (CTRP9) is a member of the adiponectin (APN) paralog CTRP family, initially identified as an adipokine modulating metabolic and cardiovascular function. Circulating CTRP9 attenuates myocardial ischemia/reperfusion (MI/R) injury, reverses post-MI remodeling, and promotes vasorelaxation in an endocrine fashion^10–15^. We and others demonstrated that CTRP9 is highly expressed in the heart, nearly 1.6-fold of circulating CTRP9 level^14,16,17^. However, myocardial capillary endothelial cell-derived CTRP9 was shown to trigger cardiomyocyte hypertrophy in a paracrine manner^17^. The discrepancy in the regulation of myocardial function by different origins of CTRP9 remains unexplained. Whether cardiac-derived CTRP9 protects against MI/R injury (and if so, by what mechanisms) is unknown.

The aims of this study were (1) to determine the role of cardiac-derived CTRP9 in MI/R injury; (2) to investigate whether cardiac-derived CTRP9 regulates myocardial dysfunction after MI/R in an autocrine or paracrine manner; (3) to elucidate the underlying mechanisms responsible for the actions of cardiac-derived CTRP9 upon MI/R injury.

## Materials and methods

All experiments were approved by the Fourth Military Medical University Committee on Animal Care. Eight- to 10-week-old C57BL/6J male mice and neonatal Sprague–Dawley rats (1–2 days old) were provided by the Experimental Animal Center of the Fourth Military Medical University (Xi’an, China). The CTRP9-knockout (KO) (on a Sprague–Dawley background) rats were generated by the K&D Gene Technology Co., Ltd (Wuhan, China). Homozygous CTRP9-KO and littermate WT rats were used in the present study. Baseline conditions of CTRP9-KO rats were recorded prior to experiments.

### In vivo siRNA-mediated cardiac CTRP9 deficiency

CTRP9 Stealth RNAi (siCTRP9, 0.8 μg/μl, Invitrogen, MSS248274) or non-specific control small interfering RNA (siRNA) (NC, Invitrogen, 12935114) pre-mixed by in vivo jet PEI (Genesee Scientific, 201-10G) were delivered via three separate intra-myocardial injections (by 32.5-gauge needle) to temporarily blanch the LV free wall as described previously^18,19^. Western blot determined knockdown efficiency 72 h later.

### In vivo lentivirus-mediated cardiac CTRP9 overexpression

Green fluorescent protein (GFP)-conjugated CTRP9 lentivirus (*LV*. CTRP9) or negative control (NC) lentivirus (NC) were injected into the left ventricle of mice (30 μl) and rats (100 μl) (Table 1). Frozen heart sections were prepared after 72 h transfection. Lentiviral location was assessed by staining with α-actin (Sigma-Aldrich, A2547) or CD31 (Boster, BA0532) antibodies (Supplemental Figs. 1B and 2A). Five sections from each heart were examined by fluorescence microscopy (Olympus, Japan). The overexpression efficiency was assessed by Western blot. Plasma CTRP9 was determined by Enzyme-Linked Immunosorbent Assay (Aviscera Bioscience, SK00081-08) to detect whether the lentivirus mediated a cardiac-specific CTRP9 overexpression (Supplemental Fig. 1D).

### Animal model of MI/R

Seventy-two hours after intra-myocardial injection of siRNA or lentivirus, mice and CTRP9-KO rats were anesthetized with 2% isoflurane. The heart was exposed by a left thoracic incision. Myocardial infarction was produced by placing a 6.0 silk suture slipknot around the left anterior descending coronary artery. After 30 min of ischemia, the slipknot was released to allow reperfusion for 3 or 24 h. Sham-operated mice/rats underwent left thoracotomy only.

### Echocardiographic analysis

Mice and CTRP9-KO rats were subjected to transthoracic echocardiography (VisualSonics VeVo 2100 Imaging System) for assessment of cardiac structure and function after 24 h reperfusion. Echocardiography was performed, and M-mode tracings were recorded.

### Evans blue/TTC staining

After 24 h reperfusion, 1.5% Evans blue (Sigma-Aldrich, E2129) was injected into the aorta. The heart was removed and frozen at −80 °C immediately. Subsequently, the heart was horizontally sectioned into 5–6 slices, and incubated with 1% 2, 3, 5-triphenyl tetrazolium chloride (TTC, Sigma-Aldrich, T8877) for 15 min at 37 °C. The left ventricular (LV) area, the area at risk (AAR), and infarct area (IA) of each section was calculated by Image J.

### TUNEL staining

Mouse and CTRP9-KO rat hearts were perfused with ice-cold phosphate-buffered saline and fixed with 4% paraformaldehyde, embedded in paraffin, and coronally sectioned (3–6 μm thick). Three to five sections from each heart were subjected to infarct area TUNEL (terminal deoxynucleotidyl transferase dUTP nick-end labeling) Staining Assay (Roche Diagnostics Corporation, 11684817910), as per the manufacturer’s protocol.

### Measurement of LDH release

Lactate dehydrogenase (LDH) activity in conditioned medium and cell lysates were determined by the LDH release assay (Institute of Jiancheng Bioengineering, A020-2). The percentage of LDH release was calculated as follows: “(A−B)/(C−B) × 100,” where A is the LDH activity in conditioned medium, B is the LDH activity in culture medium (without cells), and C is the LDH activity in cell lysates, as per the manufacturer’s protocol.

### Measurement of caspase-3 activity

Caspase-3 activity was measured via a fluorometric kit (BD Biosciences, 556574). Briefly, heart tissue or cultured cardiomyocytes were lysed on ice. The supernatant was collected. The reactions were performed in assay buffer containing 10 mM dithiothreitol (DTT) and 50 µg proteins. The fluorescence emission of the 7-amino-4-trifluoromethyl-coumarin (AFC) was measured via Spectra Max-Plus microplate spectrophotometer (Molecular Devices, excitation wavelength, 400 nm; emission wavelength, 505 nm). Caspase-3 activity was expressed as nmol AFC/h/mg protein.

### Co-immunoprecipitation

The LV tissue lysate was incubated with pre-washed Dynabeads^®^ Protein G (Invitrogen, 10003D) or Protein A (Invitrogen, 10006D) at 4 °C for 2 h. Beads were magnetically collected against the vessel wall. The supernatant was then incubated with normal immunoglobulin G (IgG), anti-CTRP9 (LifeSpan Biosciences, Inc., LS-C373857), or anti-calreticulin (CRT) (Santa Cruz, sc-373863) primary antibodies together with 15 μl pre-washed Dynabeads^®^ Protein G or Protein A at 4 °C overnight. The immunoprecipitated proteins were released from the beads using elution buffer, and mixed with a loading buffer containing 100 mM DTT. Samples were boiled and analyzed by Western blot.

### Analysis of colocalization by microscopy

Control and post-simulated ischemia/reperfusion (SI/R) neonatal rat cardiomyocytes (NCMs) were fixed by 4% paraformaldehyde, and stained with rabbit anti-CTRP9 (LifeSpan Biosciences, Inc., LS-C373857) and mouse anti-CRT (Abcam, ab22683) primary antibodies at 4 °C overnight. After washing, cells were stained with Alexa Fluor 488-conjugated goat anti-rabbit and Alexa Fluor 594-conjugated goat anti-mouse secondary antibodies (Invitrogen, A-11008 and R37121). Fluorescent images were obtained by laser scanning confocal microscopy (Fluo View TM FV 1000, Olympus).

### Plasma membrane protein extraction

Plasma membrane proteins were extracted by Plasma Membrane Protein Extraction Kit (Fisher Scientific, P503) via methods slightly modified from the manufacturer’s protocol. Briefly, heart tissues (20–30 mg) pre-mixed by 500 µL Buffer A were mechanically lysed by Dounce homogenizer. After centrifugation, the supernatant was transferred into a fresh microcentrifuge tube and centrifuged at 100,000 × *g*, 4 °C for 1 h to extract cytosolic proteins. The pellet was mixed with 500 µL Buffer B and incubated at 4 °C for 30 min. The total membrane protein fractions were gathered after 6000 × *g* centrifugation at 4 °C for 10 min. Organelle membrane proteins were extracted after 7800 × *g* centrifugation at 4 °C for 20 min, while plasma membrane proteins were extracted after additional 16,000 ×*g* centrifugation at 4 °C for 30 min.

### Simulated ischemia/reperfusion

Normal culture medium was replaced by Hanks' balanced salt solution (Gibco, 14175079). NCMs or C166 mouse embryonic yolk sac endothelial cells (MECs, purchased from ATCC) were placed in a Napco 8000WJ hypoxia (1% O_2_, 5% CO_2_, 94% N_2_) incubator (Thermo Fisher Scientific, Inc.). After 12 h of hypoxia–hypoglycemic culture, cells were bathed again in normal culture medium, and incubated for 3 or 6 additional hours in a normal CO_2_ incubator.

### Cell culture and treatments

Isolated NCMs were cultured in Dulbecco's modified Eagle's medium/F12 (Gibco, 11320033) containing 10% fetal bovine serum (Gibco, 10438026) to ~80% confluence (~5 × 10^4^ cells) as previously described^20^. Five different lentiviruses (Table 1) were used, including two NC lentivirus (GFP-conjugated or red fluorescent protein (RFP)-conjugated), GFP-conjugated CTRP9 lentivirus (*LV*. CTRP9) and RFP-conjugated lentivirus carrying CRT-short hairpin RNA (shRNA) (shCRT), or AdipoR1-shRNA. Cardiomyocytes were infected at ~100 multiplicity of infection for 24 h in the presence of 5 μg/ml Polybrene^21,22^. Cells were then bathed in normal culture medium. Stably transfected cells were selected via GFP and RFP markers after 72 h transfection. The transfective efficiency was determined by Western blot. To investigate whether autocrine CTRP9 from cardiomyocytes contributed to SI/R injury, NCMs were infected with *LV*. CTRP9 or NC for 4 h. NCMs were then cultured for 48 h without serum, after the addition of CTRP9 antibody (LifeSpan Biosciences, Inc., LS-C373857) or IgG (Cell Signaling Technology, Inc., #2729). To investigate the role of cardiac-derived CTRP9 in PKA activation, a PKA-specific inhibitor H89 (20 μM, Sigma-Aldrich, 371963-M) was employed.

**Table 1:** Lentivirus information

Gene	Lentivirus type	Sequence (5′–3′)	Titer
*Negative control (NC*)	LV5 (GFP&Puro)	TTCTCCGAACGTGTCACGT	1 × 10^8^ Tu/ml
*Negative control* *(NC*)	LV10 (RFP&Puro)	TTCTCCGAACGTGTCACGT	1 × 10^8^ Tu/ml
*C1qtnf9 mus*	LV5 (GFP&Puro)	Full sequence (NM_001191891.1)	1 × 10^9^ Tu/ml
*C1qtnf9 Rat*	LV5 (GFP&Puro)	Full sequence (NM_183175.4)	1 × 10^9^ Tu/ml
*Calr-Rat-429*	LV10 (RFP&Puro)	GCATGGAGACTCAGAATATAA	1 × 10^8^ Tu/ml
*Adipor1-Rat-1294*	LV10 (RFP&Puro)	GGAATTCCGTTACGGCCTAGA	1 × 10^8^ Tu/ml

All lentivirus were produced by Shanghai GenePharma Co., Ltd

### Western blot analysis

Mouse and rat LV tissue was harvested and lysed. Protein concentrations were determined by BCA Protein Assay Kit (Thermo Fisher Scientific, Inc., 23227). Proteins were separated by electrophoresis and transferred to polyvinylidene fluoride membranes. The membranes were blocked in Tris-buffered saline containing Tween-20 (pH 7.6) and 5% nonfat dry milk for 2 h, and subsequently incubated overnight at 4 °C with primary antibodies to the following proteins: Bcl-2 (#3498), Bax (#5023), GRP78 (#3177), caspase-12 (#2202), PKA (#4782), p-PKA (Thr197), AMPK (#2532), p-AMPK (Thr172, #2531), CREB (#9197), p-CREB (Ser133, #9198), Akt (#2967), p-Akt (Thr308, #13038), ERK1/2 (#4696), p-ERK1/2 (Thr202/Tyr204, #9106), Na^+^-K^+^-ATPase α1 (#23565), β-actin (#8457) (all from Cell Signaling Technology, Inc.), CTRP9 (LifeSpan Biosciences, Inc., LS-C373857), CRT (Abcam, ab22683 and Santa Cruz, sc-373863), AdiopR1 (Abcam, ab126611), Calnexin (Santa Cruz, sc-23954), KDEL ER marker (Santa Cruz, sc-58774), and GAPDH (CMCTAG, Inc., AT0002). After washing, the membranes were probed with appropriate secondary antibodies (Zhongshan Company, ZB-2301, ZB-2305) at room temperature for 90 min. Protein bands were detected by Bio-Rad Imaging System (Hercules), and normalized to β-actin or GAPDH.

### Statistical analysis

All values in the text and figures are presented as the mean ± standard error of mean (SEM) of *n* independent experiments. The data were analyzed using GraphPad Prism 6 statistic software (La Jolla, CA, USA). Data were subjected to *t* test (two groups) or one-way analysis of variance (ANOVA) (three or more groups). Data of CTRP9-KO rat experiments were determined by two-way ANOVA followed by post hoc test with Holm adjustment. *P* values of <0.05 (two-sided) were considered to be statistically significant.

## Results

### Cardiac-derived CTRP9 deficiency aggravates, while its overexpression ameliorates, LV dysfunction after MI/R

**Fig. 1:**
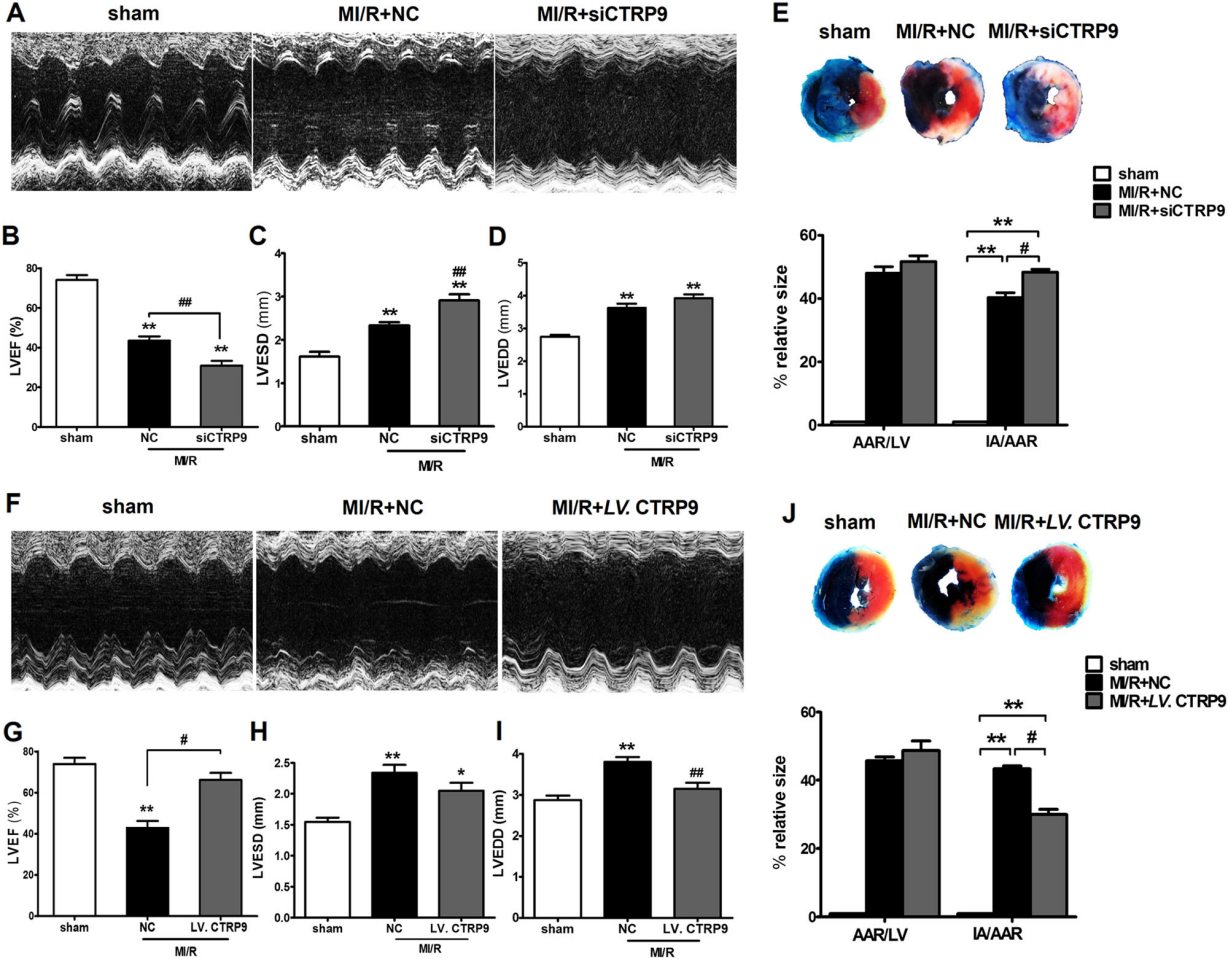
Modulation of cardiac-derived CTRP9 affects left ventricular dysfunction and myocardial infarct size after MI/R. **a**–**d** Effect of cardiac CTRP9 deficiency upon left ventricular ejection fraction (LVEF), left ventricular end-systolic diameter (LVESD), and left ventricular end-diastolic diameter (LVEDD), determined by echocardiography. **e** TTC/Evans blue double staining. The left ventricular (LV) area, the area at risk (AAR), and ischemia area (IA) were measured. **f**–**i** Effect of cardiac CTRP9 overexpression upon LVEF, LVESD, and LVEDD. **j** Effect of cardiac CTRP9 overexpression upon myocardial infarct size. Data are presented as mean ± SEM. *P *<* 0.05, **P *<* 0.01 vs. sham group; ^#^*P* < 0.05, ^##^*P* *<* 0.01 vs. MI/R + NC group. *N* = 8–12

To determine the effects of cardiac-derived CTRP9 in the setting of MI/R injury, we first utilized CTRP9 siRNA (siCTRP9) to knock down its expression in the mouse heart (Supplemental Fig. 1A). Compared to MI/R + NC group, cardiac CTRP9-deficient mice manifested lower LV ejection fraction (LVEF) with an enlarged LV end-systolic diameter following MI/R (Fig. 1a–d). Inhibition of cardiac CTRP9 increased mice myocardial infarct size with 20.4% upregulation of the IA to AAR ratio (*P* < 0.05, Fig. 1e). To confirm the role of cardiac-derived CTRP9 in MI/R injury, cardiac CTRP9 was specifically overexpressed (Supplemental Fig. 1B and 1C) without altering plasma CTRP9 level (Supplemental Fig. 1D). Cardiac CTRP9 overexpression increased animal LVEF and reduced LV end-diastolic diameter after MI/R (Fig. 1f–i). Meanwhile, cardiac CTRP9 overexpression mice manifested smaller myocardial infarct size (*P* < 0.05, Fig. 1j). Together, these data suggest that cardiac-derived CTRP9 directly protects against MI/R injury.

**Fig. 2:**
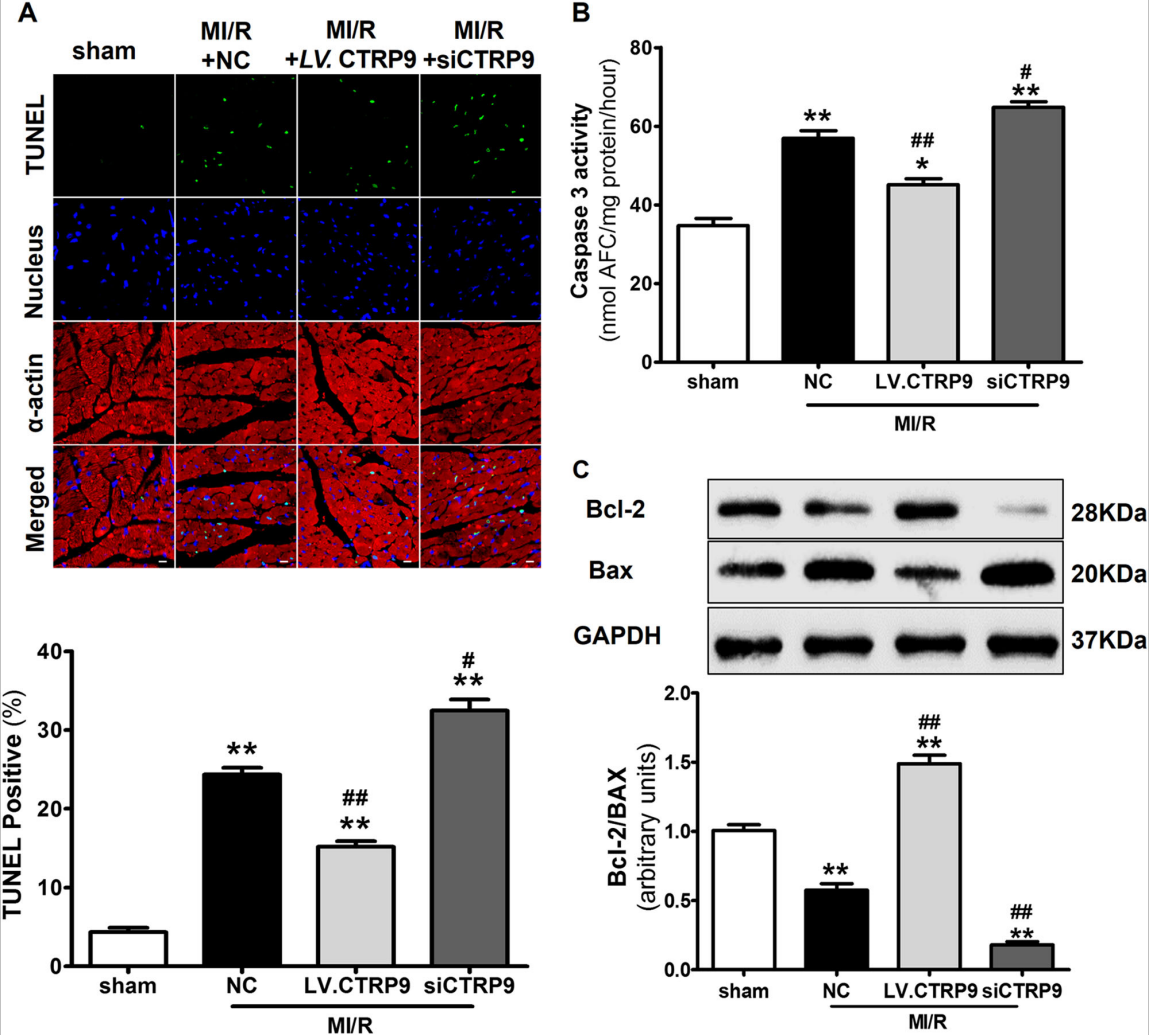
Cardiac-derived CTRP9 inhibits myocardial apoptosis subjected to MI/R injury. **a** TUNEL staining. Bar = 20 μm. **b** Caspase-3 activity. **c** Western blot analysis of Bcl-2 and Bax expression. Data are presented as mean ± SEM. *P *<* 0.05, **P *<* 0.01 vs. sham group; ^#^*P* < 0.05, ^##^*P* *<* 0.01 vs. MI/R + NC group. *N* = 5–8

### Cardiac-derived CTRP9 inhibits myocardial apoptosis after MI/R

We next assessed myocardial apoptosis in MI/R injury after genetic manipulation of cardiac CTRP9 expression. TUNEL staining results revealed that cardiac CTRP9 overexpression inhibited (*P* < 0.01), while its deficiency increased, cardiomyocyte apoptosis (*P* < 0.05) (Fig. 2a). Caspase-3 activity manifested similar changes following cardiac CTRP9 modulation (Fig. 2b). Furthermore, cardiac CTRP9 overexpression activated anti-apoptotic signaling via the increase of the Bcl-2 to Bax ratio, which was suppressed in cardiac CTRP9-deficient mice (Fig. 2c). Together, these data indicate that cardiac-derived CTRP9 exerts anti-apoptotic actions after MI/R.

### Autocrine CTRP9 of cardiomyocyte origin protects against SI/R-induced apoptosis

**Fig. 3:**
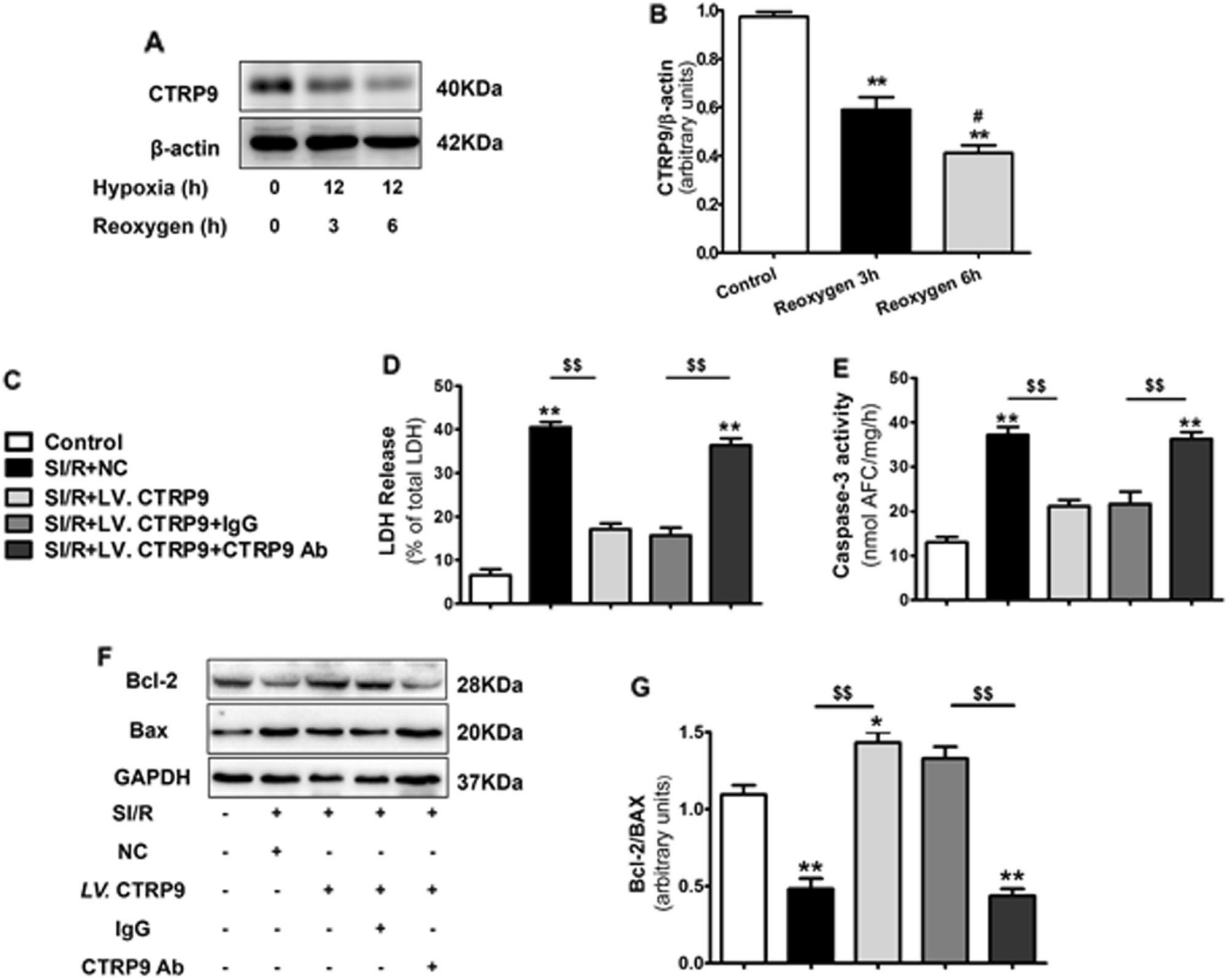
Autocrine CTRP9 of cardiomyocyte origin protects against SI/R-induced apoptosis. **a**, **b** CTRP9 expression in NCMs after hypoxia for 12 h followed by reoxygenation for 3 and 6 h. **c**–**e** Cardiomyocytes were infected with negative control (NC) or CTRP9 lentivirus (*LV*. CTRP9) and treated with CTRP9 antibody (Ab) or control IgG. Cell apoptosis were determined by LDH release and caspase-3 activity. **f**, **g** Western blot analysis of Bcl-2 and Bax expression. Data are presented as mean ± SEM. *P < 0.05, **P *<* 0.01 vs. control group; ^#^*P* < 0.05 vs. Reoxygen 3 h group; ^$$^*P* *<* 0.01 between the two groups connected by line. *N* = 3–6

To identify the origin of CTRP9 (autocrine vs. paracrine) responsive to SI/R injury, we analyzed CTRP9 expression in NCMs and MECs under SI/R. The level of CTRP9 in MECs was unchanged after SI/R (*P* = 0.09, Supplemental Fig. 2B). However, the CTRP9 expression in NCMs was decreased after 12 h hypoxia followed with 3 h reoxygenation (*P* < 0.01), reaching a lower point at 42.3% as reoxygenation time increased to 6 h (*P* < 0.01, Fig. 3a, b). NCMs were infected with *LV*. CTRP9 (Supplemental Fig. 1E and 1F) in the presence of CTRP9 antibody or control IgG. SI/R-induced LDH release and caspase-3 activity were significantly reduced by CTRP9 overexpression in NCMs (*P* < 0.01, respectively, Fig. 3c–e). Moreover, cardiac CTRP9 overexpression approximately tripled the Bcl-2 to Bax ratio (Fig. 3c, f, g). These effects were abolished by CTRP9-neutralizing antibody (*P* < 0.01, respectively, Fig. 3c–f). Together, these data suggest that cardiomyocyte-derived CTRP9 inhibits SI/R-induced apoptosis in an autocrine manner.

### The CTRP9–CRT association is responsible for the anti-apoptotic actions of cardiac-derived CTRP9

**Fig. 4:**
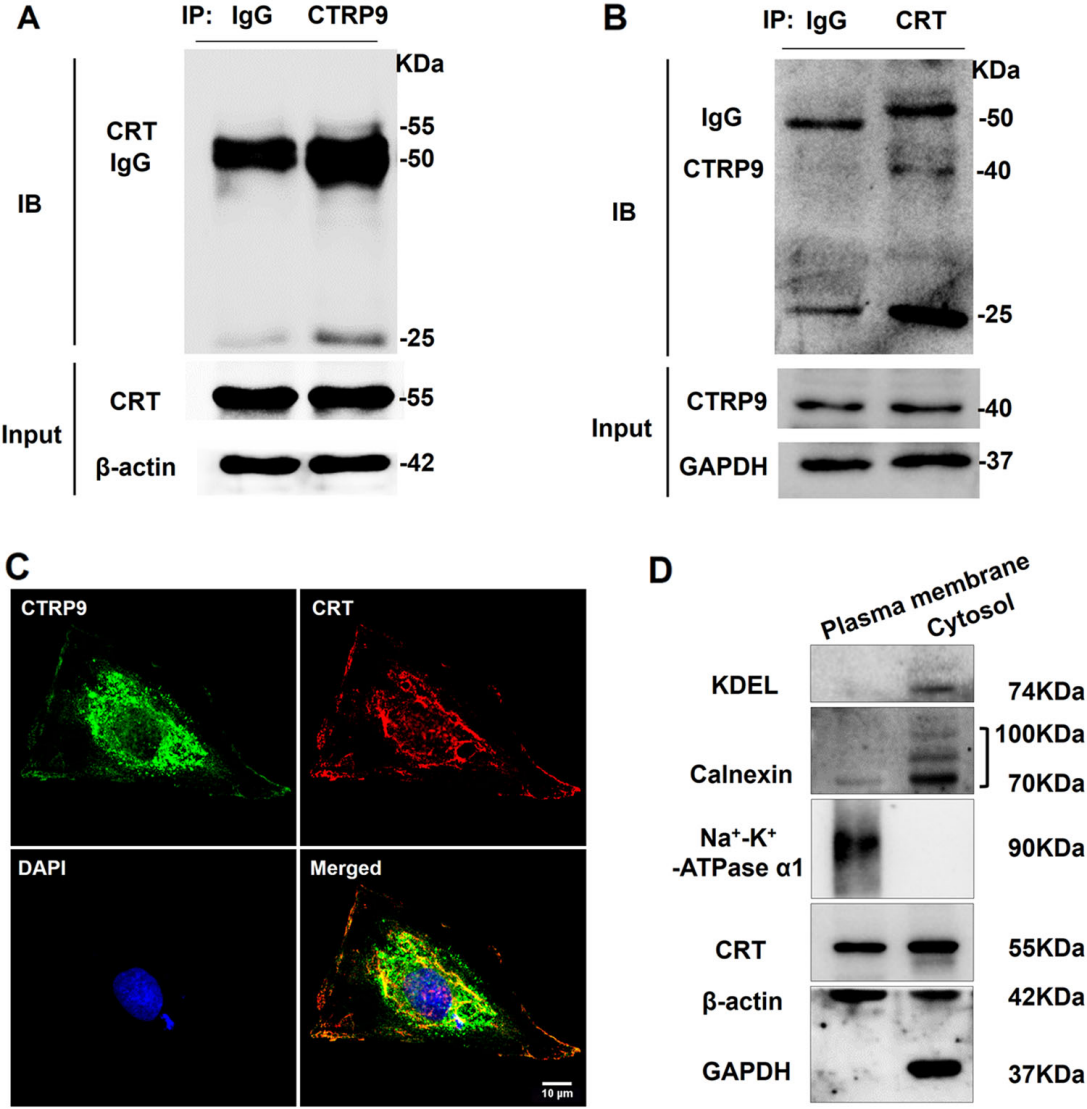
CTRP9 binds with CRT on the cell surface and in the cytoplasm of cardiomyocyte. **a** Co-immunoprecipitation (Co-IP) analysis of CRT (55 kDa band) with anti-CTRP9 antibody in left ventricular tissue lysate. **b** Co-IP analysis of CTRP9 (40 kDa band) with anti-CRT antibody in left ventricular tissue lysate. **c** Fluorescent images of the colocalization of CTRP9 and CRT in neonatal rat cardiomyocytes (NCMs) exposed to SI/R injury. Green for CTRP9, red for CRT, and blue for DAPI. Bar = 10 μm. **d** Detection of surface exposure of CRT in mouse heart tissue during MI/R injury, determined by Western blot analysis

As previous reports demonstrate circulating CTRP9 protects against MI/R injury via an AdipoR1-dependent mechanism, we determined cardiac AdipoR1 expression. AdipoR1 levels were unchanged under cardiac CTRP9 modulation (Supplemental Fig. 3A). Instead, levels of endoplasmic reticulum (ER) stress marker GRP78 were elevated concomitantly with caspase-12 after MI/R. Cardiac CTRP9 overexpression suppressed, whereas its deficiency increased, GRP78 and caspase-12 expression (Supplemental Fig. 3B–D). Notably, co-immunoprecipitation (Co-IP) results demonstrated that the ER molecular chaperone CRT immunoprecipitated with anti-CTRP9 antibody in the LV tissue lysate (Fig. 4a, 55 kDa band) and vice versa (Fig. 4b, 40 kDa band). The colocalization of CTRP9 and CRT in the cytoplasm of normal NCMs was observed via double staining of CTRP9 (green) and CRT (red) via confocal microscopy (Supplemental Fig. 3E), demonstrating that CRT binds to CTRP9 in the cardiomyocyte. In NCMs subjected to SI/R injury (12 h hypoxia followed by 3 h reoxygenation), a portion of cytosolic CRT migrated into the cell surface and combined with CTRP9 (Fig. 4c). The plasma membrane CRT was also detected in mouse heart tissue after MI/R injury (30 min ischemia followed by 3 h reperfusion, Fig. 4d).

**Fig. 5:**
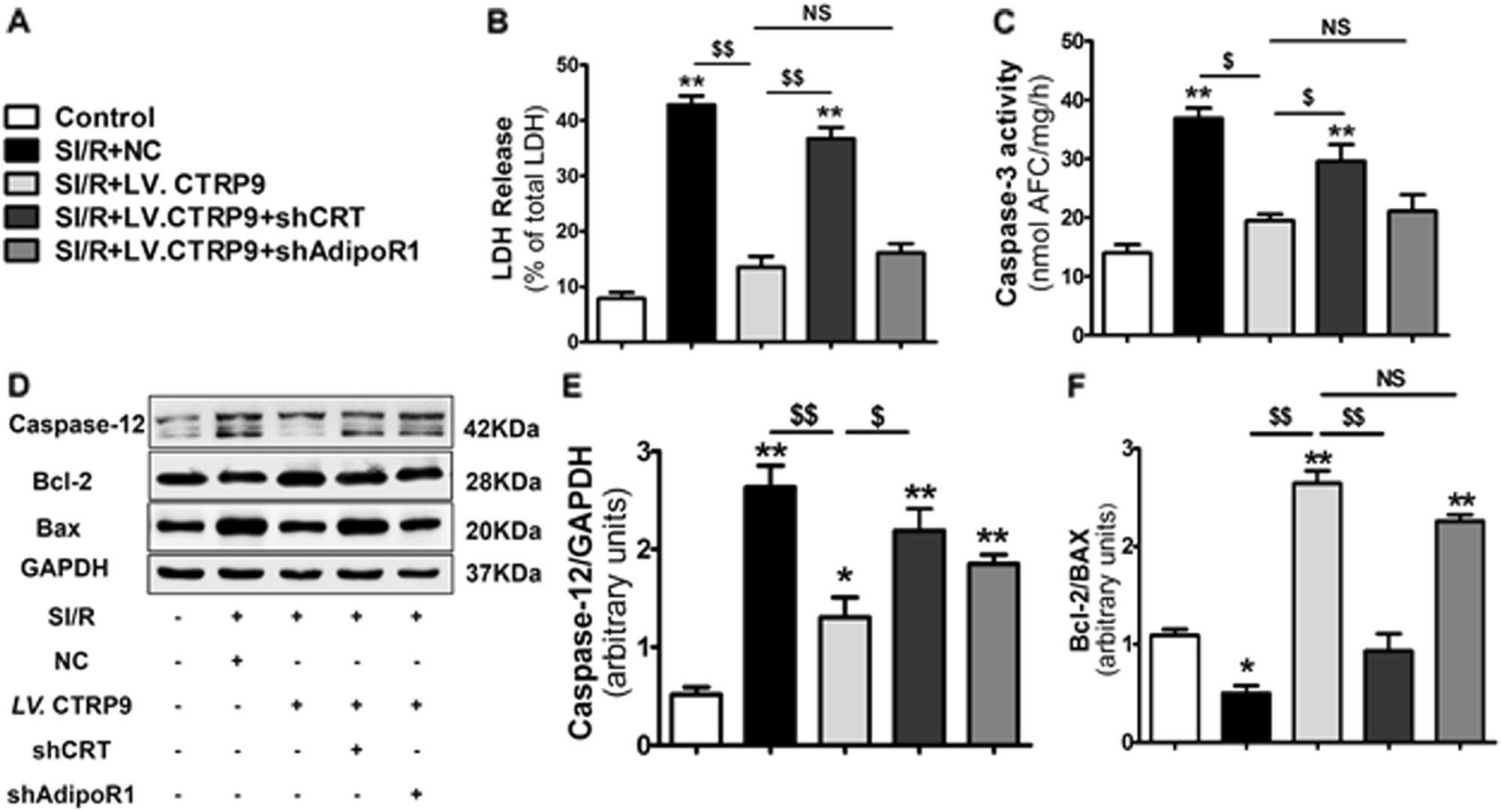
CTRP9–CRT association is responsible for the anti-apoptotic actions of cardiac-derived CTRP9. **a** Effect of CRT or AdipoR1 deficiency on LDH release. **b** Effect of CRT or AdipoR1 deficiency on caspase-3 activity. **c**–**e** Western blot analysis of caspase-12, Bcl-2, and Bax expression. Data are presented as mean ± SEM. *P < 0.05, **P *<* 0.01 vs. control group; ^$^*P* *<* 0.05, ^$$^*P* *<* 0.01 between the two groups connected by line. *N* = 3–6

To further confirm whether CRT or AdipoR1 was involved in autocrine CTRP9 anti-apoptotic actions, lentivirus were utilized to knockdown CRT and AdipoR1 expression in NCM (Supplemental Fig. 4A–C). CRT deficiency blunted cardiac-derived CTRP9’s response to LDH release (*P* < 0.01, Fig. 5a, b) and caspase-3 activity (*P* < 0.05, Fig. 5a, c). Moreover, CRT deficiency blunted cardiac-derived CTRP9’s regulation of ER stress-related apoptosis signaling pathway, demonstrated by increased caspase-12 expression and decreased Bcl-2 to Bax ratio (Fig. 5a, d–f). However, AdipoR1 deficiency had no significant effects upon cardiac-derived CTRP9’s anti-apoptotic actions (Fig. 5a–f). Together, these results suggest that cardiac-derived CTRP9 protects against cardiomyocyte apoptosis after SI/R via CRT binding.

### CTRP9–CRT activates PKA–CREB axis to inhibit cardiomyocyte apoptosis

**Fig. 6:**
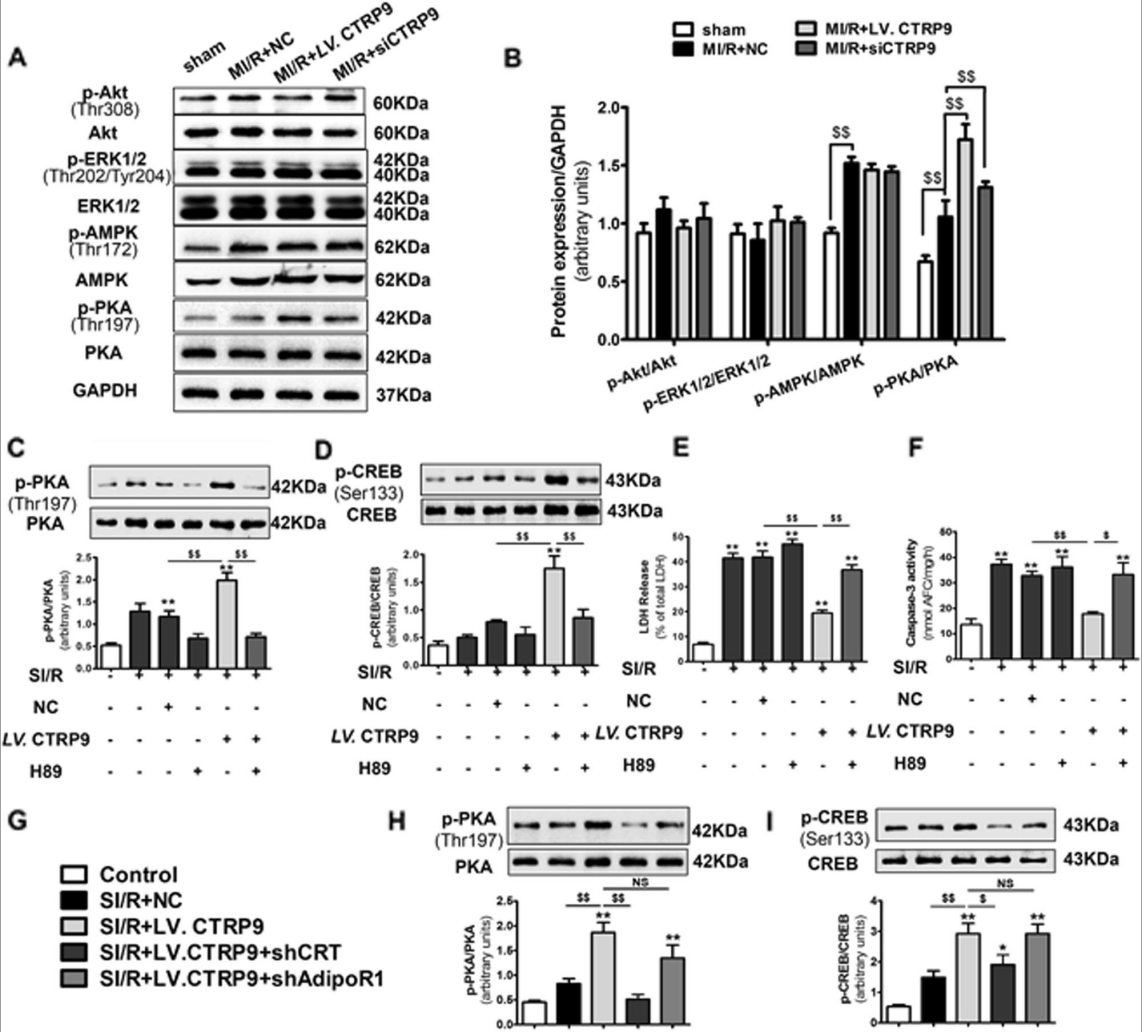
The PKA–CREB axis acts as the downstream of CTRP9–CRT interaction in MI/R injury. **a**, **b** Western blot analysis of PKA, AMPK, ERK1/2, and Akt phosphorylation in vivo. **c** H89 administration inhibits PKA activation in vitro. **d** Effect of PKA inhibition upon CREB phosphorylation. **e**, **f** Effect of PKA inhibition upon LDH release and caspase-3 activity. **g**–**i** Effect of CRT or AdipoR1 deficiency on PKA and CREB activation. Data are presented as mean ± SEM. *P *<* 0.05, **P *<* 0.01 vs. control group; ^$^*P* *<* 0.05, ^$$^*P* *<* 0.01 between the two groups connected by line. *N* = 3–6

To explore the downstream mechanism responsible for CTRP9–CRT interaction against apoptosis, we analyzed the activation of several pro-survival signaling pathways. Cardiac CTRP9 overexpression significantly activated PKA phosphorylation at Thr197 (*P* < 0.01, Fig. 6a, b). However, other signaling pathways, including AMPK, ERK1/2, and Akt, typically activated by circulating CTRP9 were not affected by cardiac CTRP9 modulation (Fig. 6a, b). Our in vitro studies consistently demonstrated that CTRP9 overexpression in NCMs significantly phosphorylated PKA and its downstream effector CREB (*P* < 0.01, respectively, Fig. 6c, d), inhibited by CTRP9 antibody (*P* < 0.05 and *P* < 0.01, respectively, Supplemental Fig. 5A–D). These results indicate that cardiac-derived CTRP9 mediates the PKA-CREB signaling pathway in an autocrine manner.

To confirm these findings, we utilized PKA-specific inhibitor, H89, in vitro. Administration of H89 virtually abolished PKA and CREB activation (*P* < 0.01, respectively, Fig. 6c, d). Meanwhile, H89 inhibited cardiac-derived CTRP9’s response to SI/R-induced LDH release (*P* < 0.01, Fig. 6e) and caspase-3 activity (*P* < 0.05, Fig. 6f). Furthermore, CRT deficiency abrogated PKA and CREB phosphorylation, while inhibition of AdipoR1 had no effects (Fig. 6g–i). Together, these data demonstrate that CTRP9–CRT initiates PKA-CREB signaling in cardiomyocyte to protect against MI/R injury.

### CTRP9-KO rats exhibit exacerbated cardiac dysfunction after MI/R, and cardiac CTRP9-specific overexpression inhibits acute myocardial injury

**Fig. 7:**
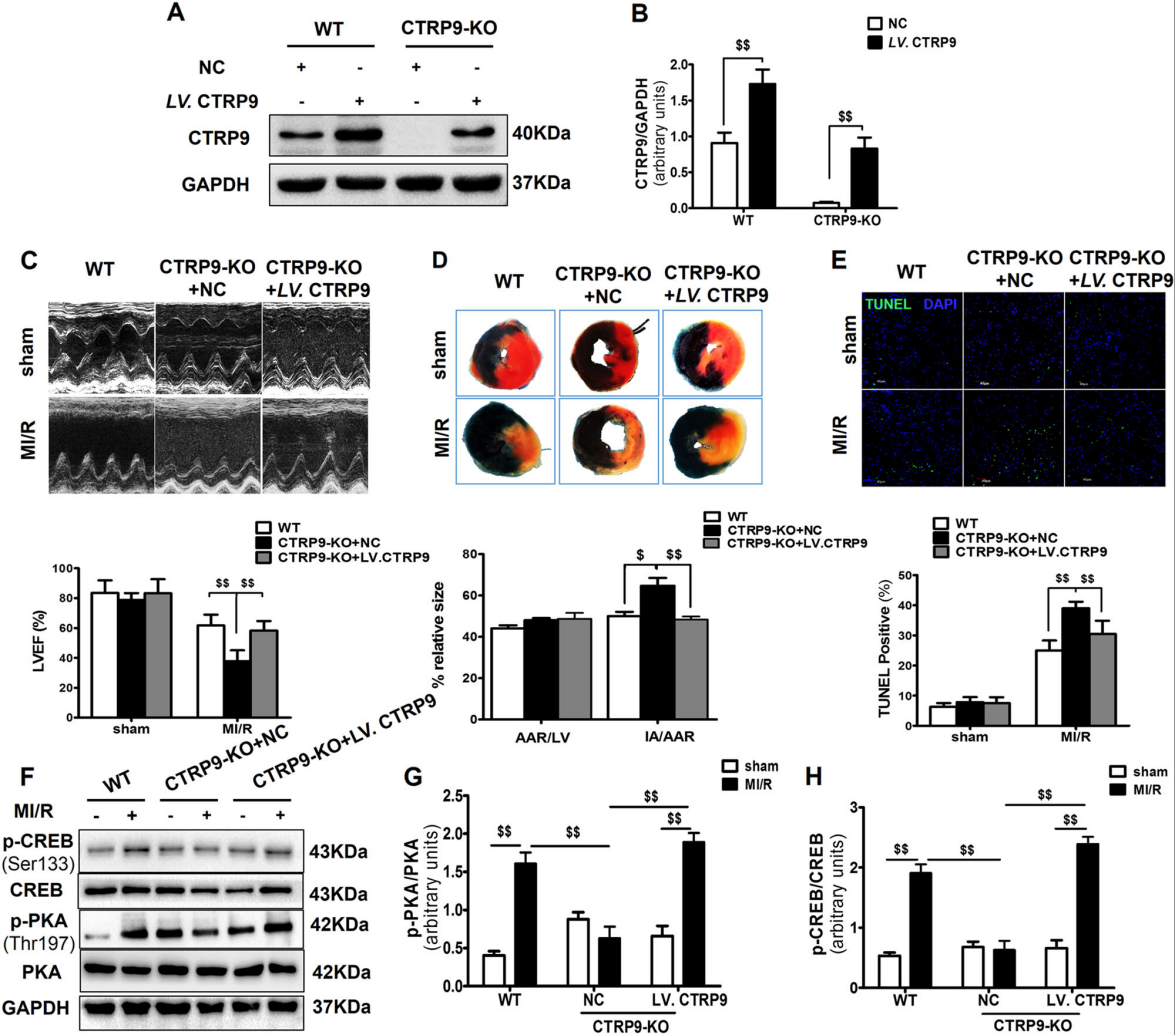
CTRP9-KO rat displays a dysfunctional phenotype following MI/R injury, and cardiac CTRP9-pecific overexpression reverses acute cardiac injury. **a**, **b** Cardiac CTRP9 expression in WT and CTRP9-KO rats receiving intra-myocardial injection of *LV*. CTRP9 or NC. **c** Representative echocardiograms with LVEF assessment of WT and CTRP9-KO rats subjected to MI/R. **d** Myocardial infarct size. **e** TUNEL staining. Bar = 40 μm. **f**–**h** Western blot analysis of PKA and CREB. Data are presented as mean ± SEM. ^$^*P* *<* 0.05, ^$$^*P* *<* 0.01 between the two groups connected by a line. *N* = 5–8

To further confirm the in vivo role of cardiac-derived CTRP9, we generated CTRP9-KO rats upon an SD background. Under baseline conditions, there were no differences between 6-week-old CTRP9-KO and littermate wild-type (WT) rats (Supplemental Table 2). CTRP9 protein was undetectable in the heart of homozygous CTRP9-KO rats. Then, a cardiac-specific expression of CTRP9 was constructed via intra-myocardial injection of *LV*. CTRP9 (Fig. 7a, b). CTRP9-KO rats exhibited lower LVEF and larger myocardial infarct size, compared with WT rats after MI/R injury. Cardiac CTRP9-specific overexpression improved rat LVEF (*P* < 0.01, Fig. 7c) and attenuated myocardial infarct size (*P* < 0.01, Fig. 7d). TUNEL staining results revealed that CTRP9 ablation significantly increased myocardial apoptosis in the AAR regions, while cardiac CTRP9-specific overexpression reduced MI/R-induced apoptosis (Fig. 7e). Furthermore, cardiac CTRP9-specific overexpression activated PKA-CREB signaling (Fig. 7f–h), consistent with in vitro results.

## Discussion

In the present study, we provide the first evidence that cardiac-derived CTRP9 exerts cardioprotection against MI/R injury in an autocrine manner. As an APN paralog, CTRP9 was initially identified as an adipokine^16^, regulating systemic metabolism and cardiovascular functions. Circulating CTRP9 level decreases after MI/R, while exogenous supplementation of recombinant globular CTRP9 (gCTRP9) reverses cardiac dysfunction. We and others have shown that CTRP9 is abundantly produced by the heart itself, nearly 1.6-fold of plasma CTRP9 level^14^ and 2-fold to 3-fold of subcutaneous fat tissue CTRP9 level^17^. However, the biologic role of cardiac-derived CTRP9 remained heretofore unclear. In the current study, we demonstrate that cardiac CTRP9 deficiency exacerbates, whereas its overexpression ameliorates, LV dysfunction and myocardial infarct size of mice in MI/R injury. CTRP9-KO rats manifest similar resistant phenotype, while cardiac CTRP9-specific overexpression reverses myocardial function and improves cell survival. Cardiac-derived CTRP9 inhibits MI/R-induced apoptosis, acting as a protective cardiokine.

A recent study demonstrated that myocardial endothelial cell-generated CTRP9 drives cardiac hypertrophy, performing a maladaptive role in cardiac function^17^. However, our in vitro results reveal that endothelial CTRP9 expression is unchanged in SI/R. Instead, CTRP9 levels in NCMs decreased during SI/R injury, while lentivirus-mediated endogenous CTRP9 overexpression inhibits SI/R-induced apoptosis. These results confirm that autocrine CTRP9 of cardiomyocyte origin at least partially responds to SI/R injury via inhibition of cell apoptosis. The divergent roles of CTRP9 of different origins may be due to different disease models activating different cell types in response. Myocardial capillary endothelial cells regulate cardiomyocyte growth, contributing to cardiac hypertrophy,^8,23^ whereas cardiomyocytes suffered from Ca^2+^ overload and mitochondrial permeability transition pore opening are involved in MI/R injury^24^.

Next, we demonstrated that cardiac CTRP9 overexpression decreases, while its deficiency increases the expression of ER stress-related apoptosis markers GRP78 and caspase-12 after MI/R. This is consistent with our previous findings in the diabetic heart^13^. Notably, we found that the ER molecular chaperone CRT, primarily located in the ER lumen under physiological conditions, migrates to the cell surface and nucleus of cardiomyocytes subjected to SI/R. Cardiac-derived CTRP9 binds to CRT both in the cytoplasm and on the cell surface of cardiomyocytes. Inhibition of CRT blunted cardiac-derived CTRP9’s anti-apoptotic actions. CRT is characterized as a molecular chaperone with functions of Ca^2+^ sensing, glycoprotein folding, and major histocompatibility complex class I assembly^25–27^. CRT translocates from the ER lumen to multiple subcellular localizations in response to stress^28–30^. It modulates cell survival via ER stress regulation^31–33^. Cytosolic CRT regulates cell adhesion^34^, and is involved in signal transduction events associated with innate immunity^35,36^. Nuclear CRT functions as a nuclear export receptor^37,38^. Cell surface CRT functions as a receptor for C1q^39^, initiating the clearance of early apoptotic cells for phagocytosis^40,41^. Karnabi et al.^42^ reported surface exposure of CRT in human fetal cardiomyocytes. In this regard, CRT may be a new receptor for cardiomyocyte-derived CTRP9. It may assist CTRP9 folding and synthesis in cardiomyocytes, and promote the internalization of autocrine CTRP9 during MI/R injury. Conversely, previous studies have shown that circulating CTRP9 regulates cardiovascular functions via AdipoR1-dependent mechanisms. However, our present results reveal that AdipoR1 expression is unchanged during cardiac CTRP9 manipulation, and inhibition of AdipoR1 does not affect cardiac-derived CTRP9’s anti-apoptotic effects after SI/R injury. A recent study identified N-cadherin as a specific receptor for CTRP9 acting upon adipose-derived mesenchymal stem cells^43^. Taking together, CTRP9 exerts cardioprotective functions at least partially via AdipoR1-independent fashion.

Finally, the present study demonstrates that the CTRP9–CRT interaction activates PKA-CREB pro-survival signaling. Previous results demonstrated that circulating CTRP9 exerts anti-inflammatory, anti-apoptotic, and cardiovascular protective actions via activation of the AdipoR1–AMPK axis^11,12,15,44,45^. However, we did not find evidence for differential activation of AMPK, ERK1/2, or Akt in response to cardiac CTRP9 modulation following MI/R injury. Instead, we observed significant phosphorylation of PKA (Thr197) and its downstream effector CREB (Ser133) in cardiac CTRP9 overexpression mice and rats, inhibited by functional neutralization of autocrine CTRP9. Meanwhile, inhibition of PKA abrogated cardiac-derived CTRP9’s anti-apoptotic actions. It might be that different CTRP9 isoforms mediate different kinase activation. When produced by the heart, CTRP9 is a full-length glycoprotein containing C1q globular domain and N-terminal domains; the CTRP9 that circulates in the plasma does so in a globular domain isoform^46^. Co-IP results revealed that CRT binds to full-length CTRP9 (40 kDa). Our previous study showed that administration of gCTRP9 for 6 weeks after MI activates PKA^10^ since sustained exogenous supplementation of gCTRP9 may increase both plasma and myocardial CTRP9 levels^43^. Furthermore, our present results suggest that CRT inhibition, not AdipoR1, blunts PKA–CREB axis activation. Cardiac-derived CTRP9 likely activates PKA-CREB signaling due to a CRT-dependent regulation of intracellular Ca^2+^ influx after MI/R injury^47,48^.

**Fig. 8:**
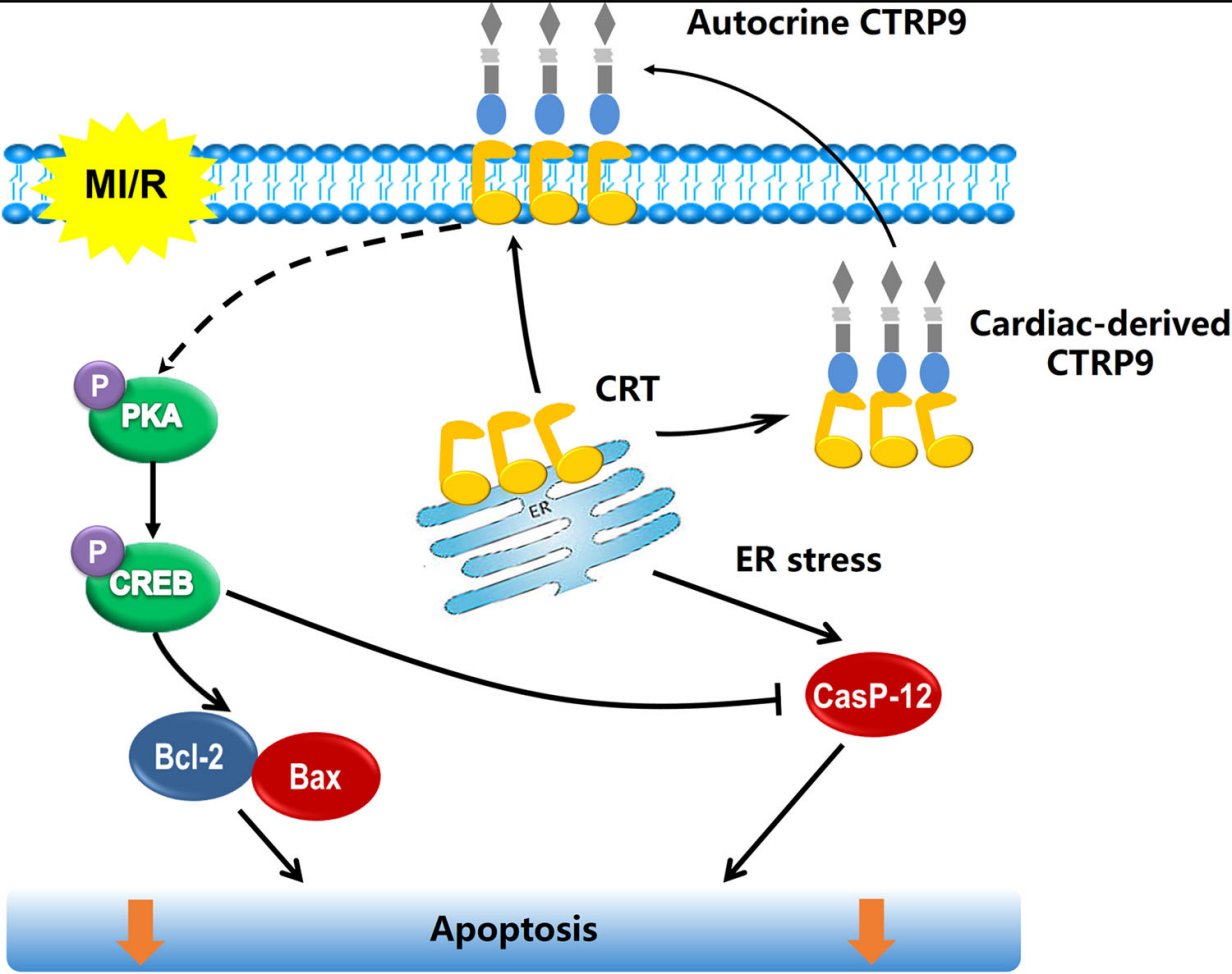
Proposed mechanism responsible for autocrine CTRP9 of cardiomyocyte origin against MI/R injury. Briefly, CTRP9 binds to ER molecular chaperone CRT in the cytoplasm of cardiomyocyte under physiological conditions. When subjected to MI/R injury, CRT translocates to cell surface and associates with autocrine CTRP9. CTRP9–CRT complex activates PKA-CREB pro-survival signaling and inhibits ER stress-related apoptotic signaling, directly protecting against MI/R injury

In conclusion, autocrine CTRP9 of cardiomyocyte origin protects against MI/R injury via CRT binding. Cardiac-derived CTRP9 activates PKA-CREB signaling and inhibits ER stress-related apoptosis signaling during MI/R injury (Fig. 8). These findings improve our understanding of CTRP9 of different cell origins in regulating cardiomyocyte apoptosis after MI/R injury, and suggest the potential value of therapeutic approaches targeting cardiac-derived CTRP9 or CRT in the treatment and prevention IHD and its complications.

## Electronic supplementary material

Supplementary Materials

Video 1

## References

[1] Hausenloy DJ, Yellon DM (2015). Targeting myocardial reperfusion injury—the search continues. N. Engl. J. Med..

[2] Mozaffarian D (2016). Heart disease and stroke statistics–2016 update: a report from the American Heart Association. Circulation.

[3] Shimano M, Ouchi N, Walsh K (2012). Cardiokines: recent progress in elucidating the cardiac secretome. Circulation.

[4] Doroudgar S, Glembotski CC (2011). The cardiokine story unfolds: ischemic stress-induced protein secretion in the heart. Trends Mol. Med..

[5] Nishikimi T, Maeda N, Matsuoka H (2006). The role of natriuretic peptides in cardioprotection. Cardiovasc. Res..

[6] Chai W, Mehrotra S, Jan Danser AH, Schoemaker RG (2006). The role of calcitonin gene-related peptide (CGRP) in ischemic preconditioning in isolated rat hearts. Eur. J. Pharmacol..

[7] Tian Y, Morrisey EE (2012). Importance of myocyte–nonmyocyte interactions in cardiac development and disease. Circ. Res..

[8] Tirziu D, Giordano FJ, Simons M (2010). Cell communications in the heart. Circulation.

[9] Younes A, Pepe S, Yoshishige D, Caffrey JL, Lakatta EG (2005). Ischemic preconditioning increases the bioavailability of cardiac enkephalins. Am. J. Physiol. Heart Circ. Physiol..

[10] Sun Y (2013). C1q/tumor necrosis factor-related protein-9, a novel adipocyte-derived cytokine, attenuates adverse remodeling in the ischemic mouse heart via protein kinase A activation. Circulation.

[11] Kambara T (2015). C1q/tumor necrosis factor-related protein 9 protects against acute myocardial injury through an adiponectin receptor I-AMPK-dependent mechanism. Mol. Cell. Biol..

[12] Kambara T (2012). CTRP9 protein protects against myocardial injury following ischemia–reperfusion through AMP-activated protein kinase (AMPK)-dependent mechanism. J. Biol. Chem..

[13] Bai S (2016). C1q/TNF-related protein 9 protects diabetic rat heart against ischemia reperfusion injury: role of endoplasmic reticulum. Stress.

[14] Su H (2013). Inhibition of CTRP9, a novel and cardiac-abundantly expressed cell survival molecule, by TNFalpha-initiated oxidative signaling contributes to exacerbated cardiac injury in diabetic mice. Basic Res. Cardiol..

[15] Zheng Q (2011). C1q/TNF-related proteins, a family of novel adipokines, induce vascular relaxation through the adiponectin receptor-1/AMPK/eNOS/nitric oxide signaling pathway. Arterioscler. Thromb. Vasc. Biol..

[16] Wong GW (2009). Identification and characterization of CTRP9, a novel secreted glycoprotein, from adipose tissue that reduces serum glucose in mice and forms heterotrimers with adiponectin. FASEB J..

[17] Appari M (2017). C1q-TNF-related protein-9 promotes cardiac hypertrophy and failure. Circ. Res..

[18] Wang Y (2010). Cardiomyocyte-derived adiponectin is biologically active in protecting against myocardial ischemia–reperfusion injury. Am. J. Physiol. Endocrinol. Metab..

[19] Sun Y (2017). Adiponectin exerts cardioprotection against ischemia/reperfusion injury partially via calreticulin mediated anti-apoptotic and anti-oxidative actions. Apoptosis.

[20] Wang Y (2012). Essential role of caveolin-3 in adiponectin signalsome formation and adiponectin cardioprotection. Arterioscler. Thromb. Vasc. Biol..

[21] Sun X (2014). p27 protein protects metabolically stressed cardiomyocytes from apoptosis by promoting autophagy. J. Biol. Chem..

[22] Liu L (2015). Up-regulated TLR4 in cardiomyocytes exacerbates heart failure after long-term myocardial infarction. J. Cell. Mol. Med..

[23] Heineke J (2012). Wag the dog: how endothelial cells regulate cardiomyocyte growth. Arterioscler. Thromb. Vasc. Biol..

[24] Turer AT, Hill JA (2010). Pathogenesis of myocardial ischemia–reperfusion injury and rationale for therapy. Am. J. Cardiol..

[25] Vassilakos A, Michalak M, Lehrman MA, Williams DB (1998). Oligosaccharide binding characteristics of the molecular chaperones calnexin and calreticulin. Biochemistry.

[26] Johnson S, Michalak M, Opas M, Eggleton P (2001). The ins and outs of calreticulin: from the ER lumen to the extracellular space. Trends Cell Biol..

[27] Camacho P, Lechleiter JD (1995). Calreticulin inhibits repetitive intracellular Ca^2+^ waves. Cell.

[28] Labriola, C. A., Conte, I. L., Lopez Medus, M., Parodi, A. J. & Caramelo, J. J. Endoplasmic reticulum calcium regulates the retrotranslocation of *Trypanosoma cruzi* calreticulin to the cytosol. *PLoS ONE* **5**, pii: e13141 (2010). 10.1371/journal.pone.0013141 PMC2950133 20957192

[29] Afshar N, Black BE, Paschal BM (2005). Retrotranslocation of the chaperone calreticulin from the endoplasmic reticulum lumen to the cytosol. Mol. Cell. Biol..

[30] Gold LI (2010). Calreticulin: non-endoplasmic reticulum functions in physiology and disease. FASEB J..

[31] Bernard-Marissal N (2012). Reduced calreticulin levels link endoplasmic reticulum stress and Fas-triggered cell death in motoneurons vulnerable to ALS. J. Neurosci..

[32] Li WH (2014). Calreticulin protects rat microvascular endothelial cells against microwave radiation-induced injury by attenuating endoplasmic reticulum stress. Microcirculation (New York, NY: 1994).

[33] Lim Y (2014). Sumoylation regulates ER stress response by modulating calreticulin gene expression in XBP-1-dependent mode in *Caenorhabditis elegans*. Int. J. Biochem. Cell Biol..

[34] Coppolino MG (1997). Calreticulin is essential for integrin-mediated calcium signalling and cell adhesion. Nature.

[35] Ogden CA (2001). C1q and mannose binding lectin engagement of cell surface calreticulin and CD91 initiates macropinocytosis and uptake of apoptotic cells. J. Exp. Med..

[36] Tarr JM (2010). A mechanism of release of calreticulin from cells during apoptosis. J. Mol. Biol..

[37] Holaska JM (2001). Calreticulin is a receptor for nuclear export. J. Cell Biol..

[38] Burns K (1994). Modulation of gene expression by calreticulin binding to the glucocorticoid receptor. Nature.

[39] Stuart GR, Lynch NJ, Day AJ, Schwaeble WJ, Sim RB (1997). The C1q and collectin binding site within C1q receptor (cell surface calreticulin). Immunopharmacology.

[40] Takemura Y (2007). Adiponectin modulates inflammatory reactions via calreticulin receptor-dependent clearance of early apoptotic bodies. J. Clin. Investig..

[41] Gardai SJ (2005). Cell-surface calreticulin initiates clearance of viable or apoptotic cells through *trans*-activation of LRP on the phagocyte. Cell.

[42] Karnabi E, Qu Y, Yue Y, Boutjdir M (2013). Calreticulin negatively regulates the surface expression of Cav1.3 L-type calcium channel. Biochem. Biophys. Res. Commun..

[43] Yan, W. et al. CTRP9 regulates the fate of implanted mesenchymal stem cells and mobilizes their protective effects against ischemic heart injury via multiple novel signaling pathways. *Circulation* **28**, 2162–2177 (2017). 10.1161/CIRCULATIONAHA.117.029557 PMC5705403 28978553

[44] Li Y, Geng X, Wang H, Cheng G, Xu S (2016). CTRP9 ameliorates pulmonary arterial hypertension through attenuating inflammation and improving endothelial cell survival and function. J. Cardiovasc. Pharmacol..

[45] Liu Q (2017). C1q/TNF-related protein 9 inhibits the cholesterol-induced vascular smooth muscle cell phenotype switch and cell dysfunction by activating AMP-dependent kinase. J Cell Mol Med.

[46] Yuan Y (2015). C1q-TNF-related protein-9, a novel cardioprotetcive cardiokine, requires proteolytic cleavage to generate a biologically active globular domain isoform. Am. J. Physiol. Endocrinol. Metab..

[47] Kusama K, Yoshie M, Tamura K, Imakawa K, Tachikawa E (2015). EPAC2-mediated calreticulin regulates LIF and COX2 expression in human endometrial glandular cells. J. Mol. Endocrinol..

[48] Brewster AL (2016). Early cardiac electrographic and molecular remodeling in a model of status epilepticus and acquired epilepsy. Epilepsia.

