# Supplementary material for: Cardiac-derived CTRP9 protects against myocardial ischemia/reperfusion injury via calreticulin-dependent inhibition of apoptosis
Source: Cell Death Dis. 2018 Jun 20;9(7):723. doi: 10.1038/s41419-018-0726-3 (PMC6010444; doi:10.1038/s41419-018-0726-3)
Supplement: Supplementary file 1 — Supplementary Materials [file 41419_2018_726_MOESM1_ESM.doc]

**Supplementary Materials**

**Table 2. Morphometric parameters of CTRP9-KO rats**

| **Parameter** | **Mean ± SEM** | | ***P value*** |
| --- | --- | --- | --- |
|  | **WT** | **CTRP9-KO** |  |
| **BW (g)** | 132.9 ± 11.50 | 139.3 ± 3.61 | 0.5547 |
| **Heart (g)** | 0.55 ± 0.073 | 0.60 ± 0.049 | 0.5872 |
| **Lung (g)** | 1.67 ± 0.360 | 1.48 ± 0.190 | 0.6110 |
| **Tibial length (cm)** | 3.43 ± 0.199 | 3.50 ± 0.137 | 0.7669 |
| **HW/BW (g/g)** | 0.005 ± 0.0005 | 0.006 ± 0.0005 | 0.1152 |
| **HW/LW (g/g)** | 0.39 ± 0.047 | 0.48 ± 0.062 | 0.3174 |
| **HW/TL (g/cm)** | 0.16 ± 0.015 | 0.17 ± 0.009 | 0.4940 |
| **Fasting glucose (mM/L)** | 4.4 ± 0.19 | 4.9 ± 0.18 | 0.0845 |

BW, body weight; HW, heart weight; LW, lung weight; TL, tibial length

**
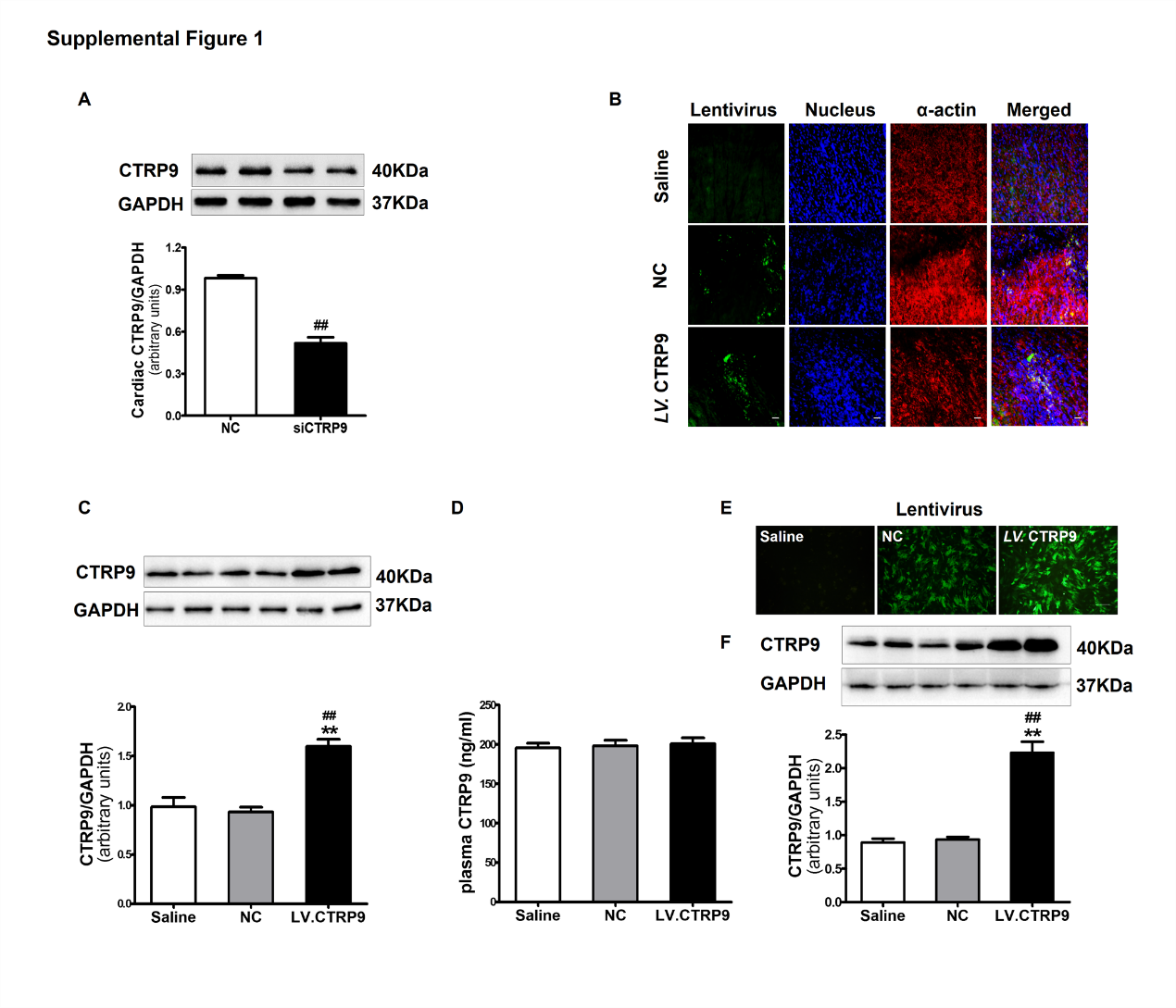
**

**Supplemental Figure 1. Genetic manipulation of cardiac CTRP9 expression.** (A) Cardiac CTRP9 knockdown efficiency in vivo, determined by Western blot. (B) Representative images of GFP-conjugated *LV.* CTRP9 or NC transfection. Bar=40 μm. (C) Efficiency of cardiac CTRP9 overexpression in vivo, determined by Western blot. (D) Plasma CTRP9 level after cardiac *LV.* CTRP9 transfection, determined by ELISA. (E) Representative images of GFP-conjugated *LV.* CTRP9 or NC in NCM after 72 hours transfection. Bar=100 μm. (F) Efficiency of cardiac CTRP9 overexpression in NCM, determined by Western blot. Data presented as mean±SEM. ^**^*P* <0.01 vs. Saline group; ^##^*P* <0.01 vs. NC group. N=3-6

**
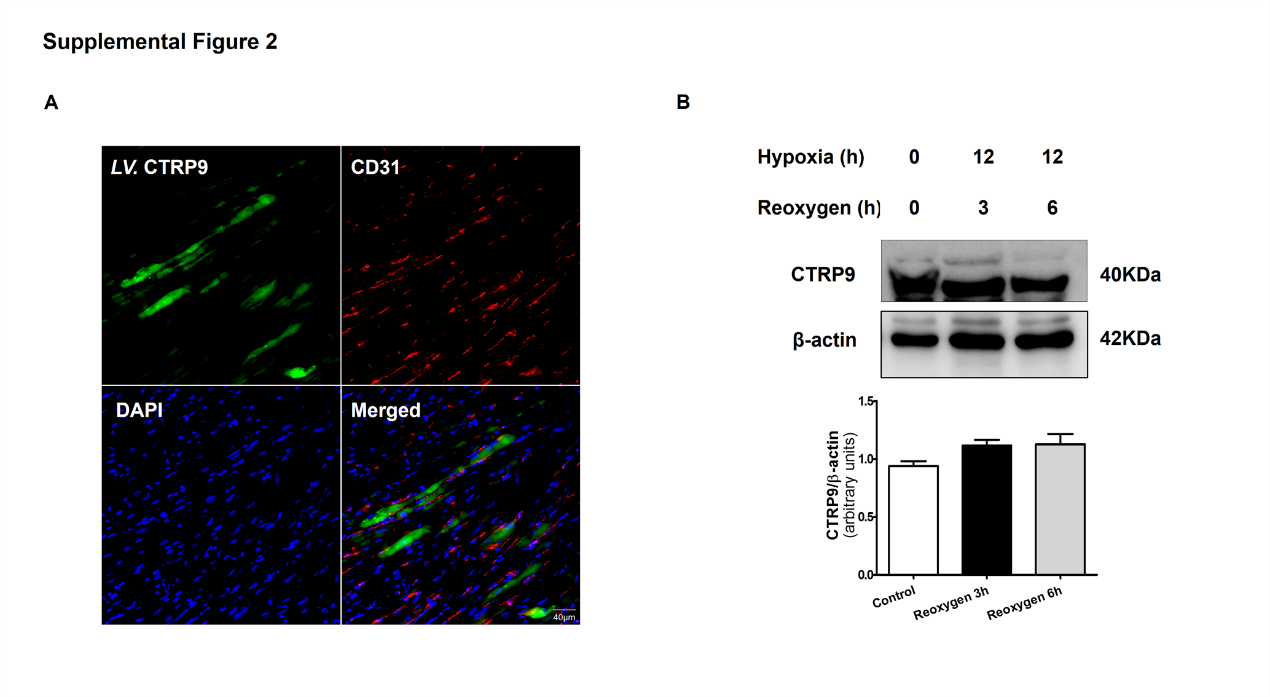
**

**Supplemental Figure 2. Cardiac CTRP9 expression in endothelial cells.** (A) Representative images of the localization of GFP-conjugated *LV.* CTRP9 and cardiac endothelial cells (stained with CD31 antibody followed by Alexa Fluor 594-conjugated second antibody). Bar=40 μm. (B) Western blot analysis of CTRP9 expression in C166 mouse embryonic yolk sac endothelial cells (MEC) after SI/R injury. Data presented as mean±SEM. N=3-6

**
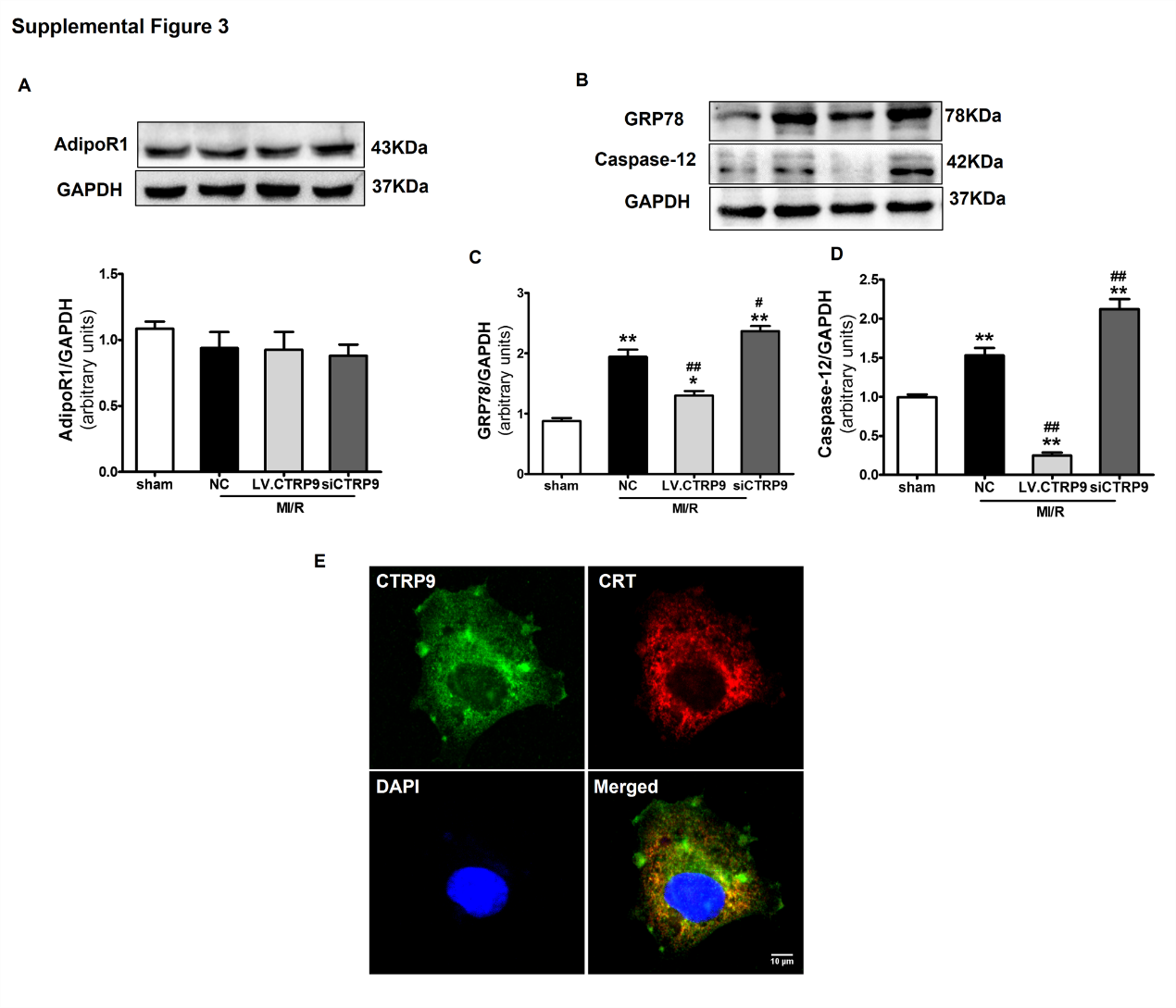
**

**Supplemental Figure 3. Effect of cardiac CTRP9 modulation upon AdipoR1 expression and ER stress markers.** (A) Western blot analysis of AdipoR1. (B-D) Western blot analysis of GRP78 and caspase-12. (E) Fluorescent images of the colocalization of CTRP9 and CRT in neonatal rat cardiomyocytes (NCM) under normal conditions. Green for CTRP9, red for CRT, and blue for DAPI. Bar=10 μm. Data presented as mean±SEM. ^*^*P* <0.05, ^**^*P* <0.01 vs. sham group; ^#^*P*<0.05, ^##^*P* <0.01 vs. MI/R+NC group. N=5-8


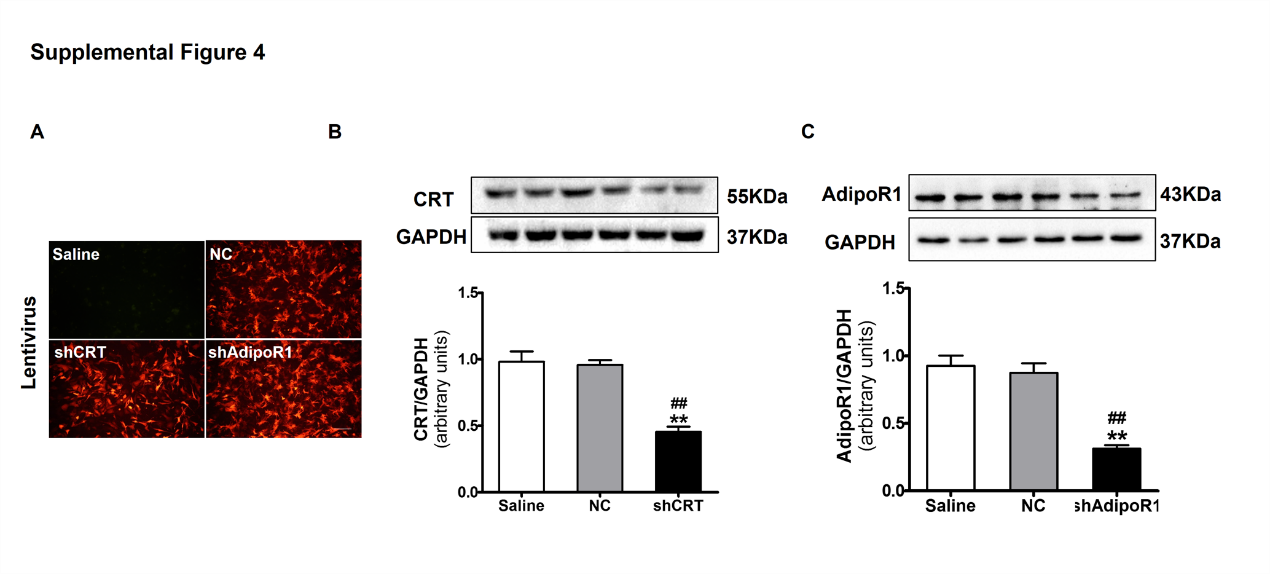


**Supplemental Figure 4. Efficiency of CRT and AdipoR1 inhibition in vitro.** (A) Representative images of RFP-conjugated lentivirus carrying CRT-shRNA (shCRT), AdipoR1-shRNA (shAdipoR1) or NC transfected into NCM for 72 hours. Bar=100 μm. (B) CRT knockdown efficiency, determined by Western blot. (C) AdipoR1 knockdown efficiency, determined by Western blot. Data presented as mean±SEM. ^**^*P* <0.01 vs. Saline group; ^##^*P* <0.01 vs. NC group. N=3-6


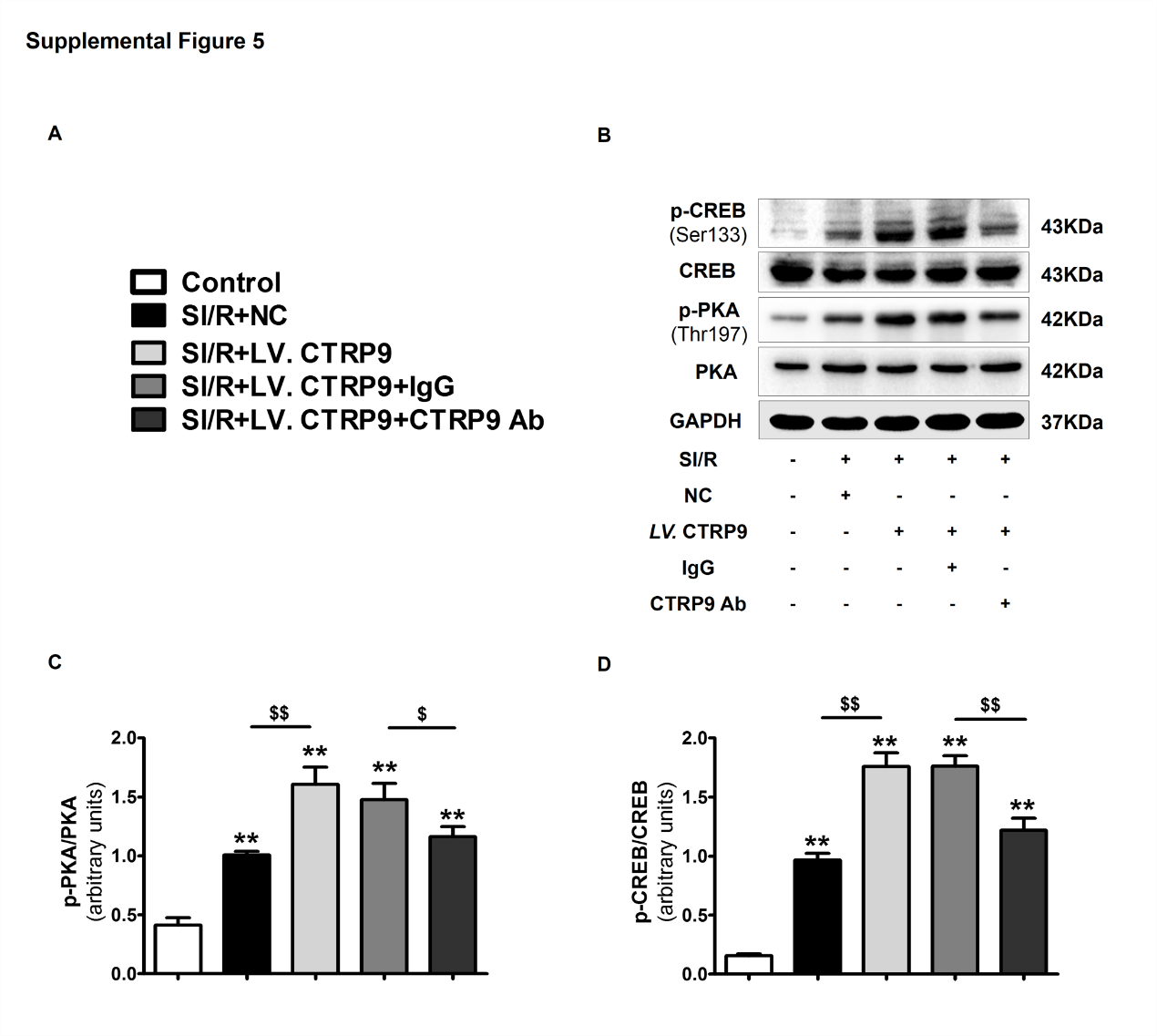


**Supplemental Figure 5. Autocrine CTRP9 of cardiomyocyte origin activates PKA-CREB axis.** (A) Group legend. (B-D) Western blot analysis of PKA and CREB phosphorylation in the presence of CTRP9 antibody or control IgG. Data presented as mean±SEM. ^**^*P* <0.01 vs. control group; ^$^*P* <0.05, ^$$^*P* <0.01 between the two groups connected by line. N=3-6

**Video 1.** Representative video of beating NCM transfected by GFP-conjugated *LV.*

CTRP9 together with RFP-conjugated shCRT for 72 hours.
